# Gross anatomy of the musculature of the thoracic limb of the aulacod

**DOI:** 10.1002/vms3.729

**Published:** 2022-01-10

**Authors:** Dieudoné Kabkia, Aklesso Ataba, Kondi Charles Agba

**Affiliations:** ^1^ Department Anatomy Histology Embryology Inter‐State School of Veterinary Sciences and Medicine of Dakar Dakar Senegal; ^2^ Department of Animal and Veterinary Sciences École Supérieure d'Agronomie Lomé Togo; ^3^ Department Anatomy Histology Embryology Inter‐State School of Veterinary Sciences and Medicine of Dakar Dakar Senegal

**Keywords:** cane rat, illustrations, limb, myology, thoracic

## Abstract

**Objective:**

The cane rat is a wild rodent appreciated for its meat. Currently domesticated, its breeding is expanding in sub‐Saharan Africa where it originates. However, the pathological problems already identified during the domestication phase of the cane rat on station still constitute a major constraint to the development of aulacodiculture. Our study aims at filling the lack of illustrations on the myology of the thoracic limb of the cane rat

**Methodology:**

We used 16 adults cane rats ranging in age from 3 to 4 years

**Results:**

The study of the different muscles of the thoracic limb reveals many similarities in the Glires (rodents and lagomorphs). However, some particularities are noteworthy. Indeed, in the cane rat, the deltoid muscle, as well as the muscles of the forearm, are very developed with its three portions, the biceps brachii and brachialis muscles are fusiform and the triceps brachii muscle has a very bulky long part (caput longum)

**Conclusion:**

These illustrations will be very effective tools for understanding the muscles of the thoracic limbs and a reliable technical support for veterinary students and practitioners.

## Graphical abstract

Our study aims at filling the lack of illustrated plates on the myology of the thoracic limb of the aulacod.

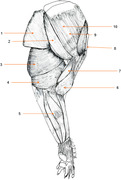

## 1 INTRODUCTION

The aulacod or cane rat, *Genus Thryonomys swinderianus*, is an animal that has been the subject of numerous studies and research in recent years; the goal of this work being the domestication of this animal (Mensah et al, 2007b). This research on aulacodiculture has progressed considerably, so that the aulacode has been raised from a hunting game in 1983 to a farmed animal since 1994. Better still, its intensive breeding in close captivity has since become a source of additional income for the agro‐aulacodic farmers. However, the pathological problems already identified, including respiratory, gastrointestinal and osteoarticular diseases, during the domestication phase of the aulacode in station (Akomedi, 1988; Alidou, 1987; Vodjo, 1986), still constitute a major constraint to the development of aulacodiculture. Knowledge of the anatomy of the aulacode is an important asset for clinicians and specialists in the pathologies of this species.

Veterinary anatomy is the science that studies the structure and morphology of animals, especially domestic animals. It seeks knowledge of the structures that make up living organisms by specifying their situations, their forms, their relationships, their functions and their particularities. Descriptive in nature, it constitutes one of the important pillars of biomedical knowledge, especially since the search for abnormalities in organs assumes that the organs of healthy animals are perfectly described and known.

However, to our knowledge, there are no scientific works and especially anatomical illustrations on the aulacode in general and on the myology of the thoracic limbs of the aulacode in particular, and yet the thoracic limb plays a very important role in the life of the aulacode, especially in the prehension of food.

Moreover, the teaching or even the transmission of veterinary anatomy requires the realisation of anatomical illustrations whose fine and sure lines reproduce almost perfectly on paper the curves and particularities of the species. It is a very effective tool for understanding anatomy and a reliable technical support for veterinary students and practitioners. It is only right that ‘the master of anatomical drawing in the twentieth century’, Dr. F.H. Netter in an essay published in 1957 recalled that anatomical illustration has three main functions: the first is that it helps the person who makes it in his or her own understanding, the second is that it helps in the transmission of information and the third is that it preserves the information for posterity (Netter, 1957). Books like *Comparative Anatomy of Domestic Mammals* (Volumes 1 to 7) by Robert Barone, *Atlas of Anatomy Veterinary* by Peter Popesko are perfect examples.

It is for this reason that our study aims at filling the lack of illustrated illustrations on the myology of the thoracic limb of the aulacode.

## 2 MATERIALS AND METHODS

### 2.1 Setting and period of study

Our study was carried out at the Laboratory of Animal Biology from 1 December 2020 to 31 May 2021.

### 2.2 Materials

#### 2.2.1 Animal material

We used 16 adults (8 males and 8 females) aulacodes from the aulacode training and breeding farm. The 16 aulacodes were old animals (more than 4 years old), died of natural causes and whose weight varies between 3 and 4 kg. It should be remembered that in the aulacodes, the sexual maturity in the male is 8 months (32 weeks) at least 2.5 kg body weight and in the female is 6½ months (26 weeks) at least 1.8 kg body weight body weight (Mensah, 2007).

#### 2.2.2 Technical laboratory equipment

The equipment used is a classic anatomy dissection kit composed of n°4 scalpel handles, dissection blades (n°21; 22; 23), pairs of curved and straight scissors, mouse tooth, hemostatic and Kocher forceps, a pair of hooks.

### 2.3 Methods

#### 2.3.1 Dissection technique

The dissection technique used was general or standard anatomical dissection technique.

#### 2.3.2 Method of obtaining the illustration

We reproduced the two faces, lateral and medial, of the different muscle segments. First, using pencils, we made drawings, from observation by eye of specimen of the different muscles on paper. Then, we traced these drawings to redraw them with black India ink pens. Finally, we scanned our drawings to obtain images. Finally, for each image, arrows were used to annotate the characteristic anatomical elements in order to facilitate the understanding of the images and with the Windows Screen Capture and Sketch Tool, the arrowed and numbered images were captured in order to avoid moving the arrows.

#### 2.3.3 Methods of anatomical description of the muscles

To carry out the description of the muscles, we were inspired by the works of Barone (1986; 2000).

## 3 RESULTS

### 3.1 Shoulder muscles

#### 3.1.1 Lateral scapular region (Figure 1)

##### Deltoid muscle

The deltoid muscle is a flat, triangular muscle, which proceeds from the scapula, laterally, by an aponeurosis all the more extended as it goes up near the dorsal edge of the bone. The deltoid muscle, in the aulacodes, has three portions: the acromial part, the clavicular part and the spinal part. The acromial part is attached to the hamatus process of the scapula and to the ventral edge of the suprahamatus process. The acromial part has a more or less triangular shape. The clavicular part is attached to the clavicle and is also triangular in shape. The spinal part is small and leaves along the scapular spine. The spinal part inserts on the deltoid tuberosity of the humerus. This muscle is the main abductor of the anatomical region of the arm.

**FIGURE 1:**
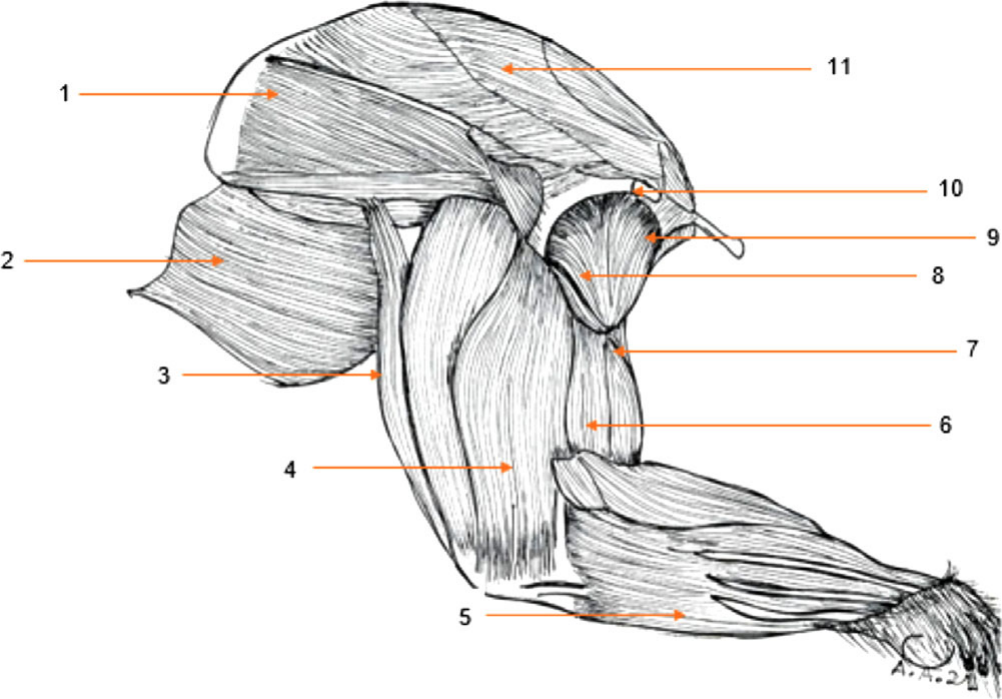
Muscles of the thoracic limb of the aulacod (right limb – lateral aspect): 1: infraspinous muscle, 2: dorsalis major muscle, 3: long head of the triceps brachii, 4: lateral head of the triceps brachii, 5: caudal antebrachial muscles, 6: brachial muscle, 7: biceps brachii muscle, 8: acromial part of the deltoid muscle, 9: clavicular part of the deltoid muscle, 10: spinal part of the deltoid muscle, 11: acromion, 12: supraspinous muscle

##### Teres minor muscle

It is small and narrow. This muscle is covered by the deltoid muscle. it originates on the caudal edge of the scapula, at the limit of the infraspinous fossa; it never goes up to the caudal angle of the bone, but its attachment always extends distally to the infraglenoid tubercle. Its terminal tendon inserts in a small special relief (tuberosity of the lesser tubercle) located on the caudal reverse of the tricipital line of the humerus, a little closer to the deltoid tuberosity.

It is an accessory of the infraspinous muscle (accompanies and helps the infraspinous muscle). It is an abductor and rotator outside the anatomical region of the arm and contributes to the contention of the scapulohumeral joint.

##### Supraspinous and infraspinous muscles

The supraspinous muscle is slightly wider and thicker than the infraspinous muscle. Each muscle occupies the fossa of the same name on the scapula. The infraspinous muscle is covered in its terminal part by the deltoid muscle. The supraspinous is the main extensor of the anatomical region of the arm while the infraspinous is an abductor muscle.

#### 3.1.2 Medial scapular region (Figure 2)

##### Subscapularis muscle

The subscapularis muscle is bulky, spread out in the subscapular fossa, against which it is moulded. It is flattened from side to side (in direction medial ‐lateral) and intermingled with tendon intersections, and it narrows and thickens towards the humerus (in direction dorsal–ventral). The terminal tendon attaches to the crest of the minor tubercle of the humerus. It allows adduction movements of the anatomical region of the arm.

**FIGURE 2:**
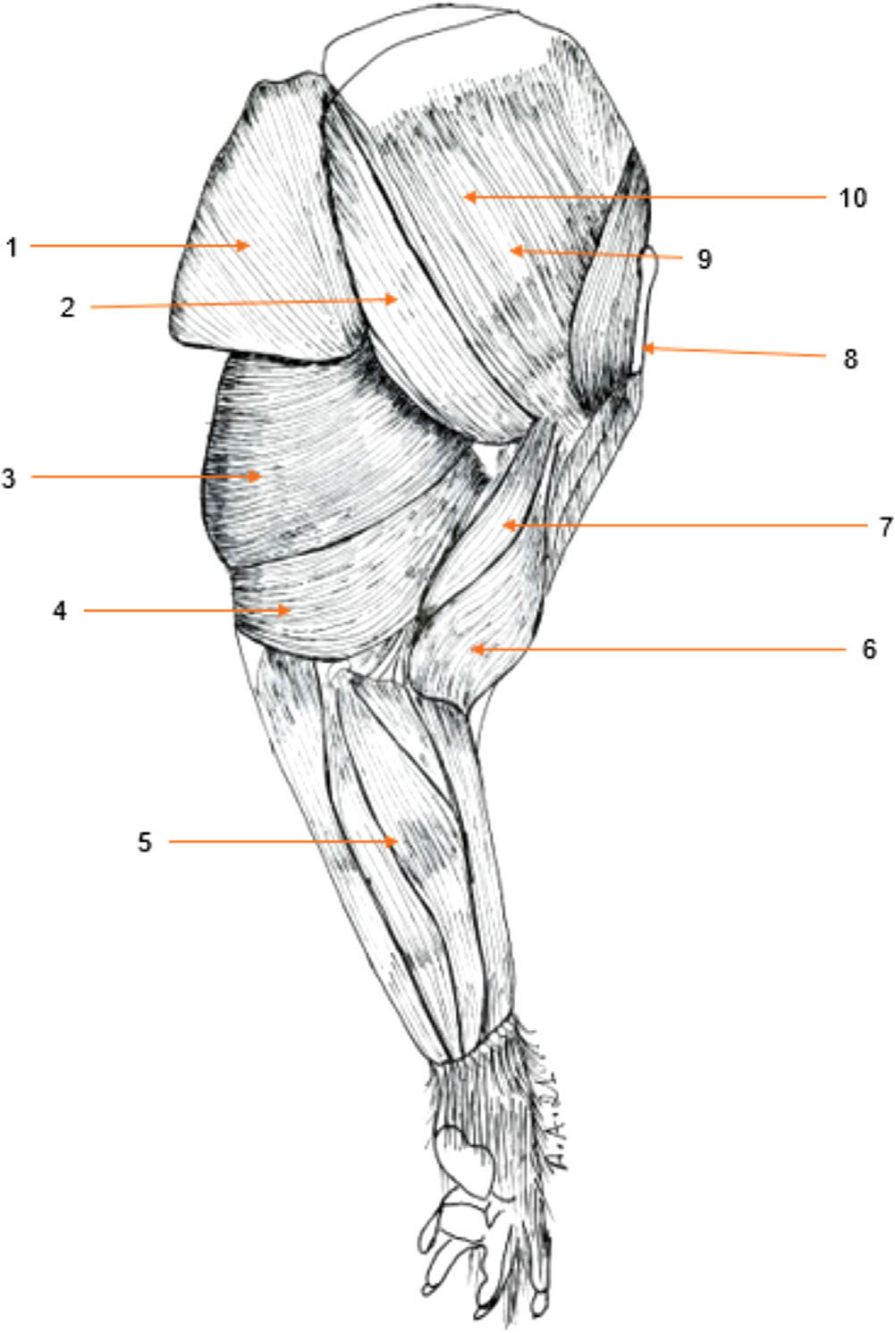
Muscles of the thoracic limb of the aulacod (left limb – medial side): 1: dorsalis major muscle, 2: teres major muscle, 3: long head of triceps brachii, 4: medial head of triceps brachii, 5: caudal antebrachial muscles, 6: biceps brachii muscle, 7: coracobrachial muscle, 8: clavicle, 9: supraspinatus muscle, 10: subscapularis muscle

##### Teres major muscle

The round muscle is large and thick. It is less voluminous than the dorsalis major to which its tendon is more or less closely united. It originates at the caudal angle of the scapula and on the adjacent part of the caudal border. It inserts on the major tuberosity on the medial aspect of the humerus. The round muscle is an adductor of the anatomical region of the arm, to which it also imparts an inward rotation; it is thus auxiliary to the dorsalis major and subscapular muscles. In synergy with the deltoid muscle, it flexes the humerus directly on the scapula.

##### Coracobrachial muscle

This long adductor muscle has a large and strong fleshy branch. It covers the end of the muscle teres major and subscapularis muscles and descends further down the humerus. The middle and upper portions can be observed. It originates at the top of the coracoid process of the scapula

The termination is on the medial side of the humerus, near that of the round and dorsalis major muscles.

### 3.2 Muscles of the arm

#### 3.2.1 Cranial brachial region (Figures 1 and 2)

##### Biceps brachii muscle

The proximal tendon, which originates on the supraglenoid tubercle, is fusiform and the muscle inserts distally on the radius with a larger oval shape. The biceps brachii muscle flexes the forearm by carrying it slightly outwards.

##### Brachial muscle

This muscle is fusiform and long on the whole caudal part of the humerus. Its fleshy part originates on the entire proximal part of the brachial groove of the humerus. The muscle originates from the proximal part of the brachial sulcus of the humerus and inserts at the medial side of the base of the olecranon. The brachialis muscle is an auxiliary of the biceps in the flexion of the forearm.

#### 3.2.2 Caudal brachial region

##### The triceps brachii muscle (Figures 1 and 2)

The caput laterale is broad; its distal half merges with the caput longum, which it covers. It originates on the entire tricipital line of the humerus, from the caudal reverse of the neck to the deltoid tuberosity and inserts on the adjacent part of the tuberosity of the olecranon.

The caput longum is a large muscle which originates at the level of the infraglenoidal tubercle of the scapula and inserts at the caudal part of the tuberosity of the olecranon.

The caput mediale is almost as long, but much narrower, relatively thick and entirely distinct from the previous two. It is dark in colour, lined caudally by a fascia that strengthens to its termination. It originates on the medial side and adjacent to the caudal side of the humeral body and inserts with the caput longum and inserts at the caudal part of the tuberosity of the olecranon.

With the help of the other parts of the triceps brachii muscle, the main function of the caput longum is to extend the forearm. The olecranon, on which it inserts, constitutes a lever arm particularly favourable to this action. When the limb is in support, the triceps open the angle of the elbow by putting in play a lever of the first kind. During the support, it acts with the same result, but by a lever of the second kind.

##### Anconeus muscle (Figure 3)

It is short, flattened and hidden. The lateral anconeal muscle is not very distinct, but there is a medial anconeal muscle. It originates at the periphery of the olecranon fossa and ascends to the midpoint of the caudal surface of the humerus and inserts at the level of the lateral reverse of the tuberosity and the cranial edge of the olecranon. It is an accessory muscle of the triceps brachii muscle.

**FIGURE 3:**
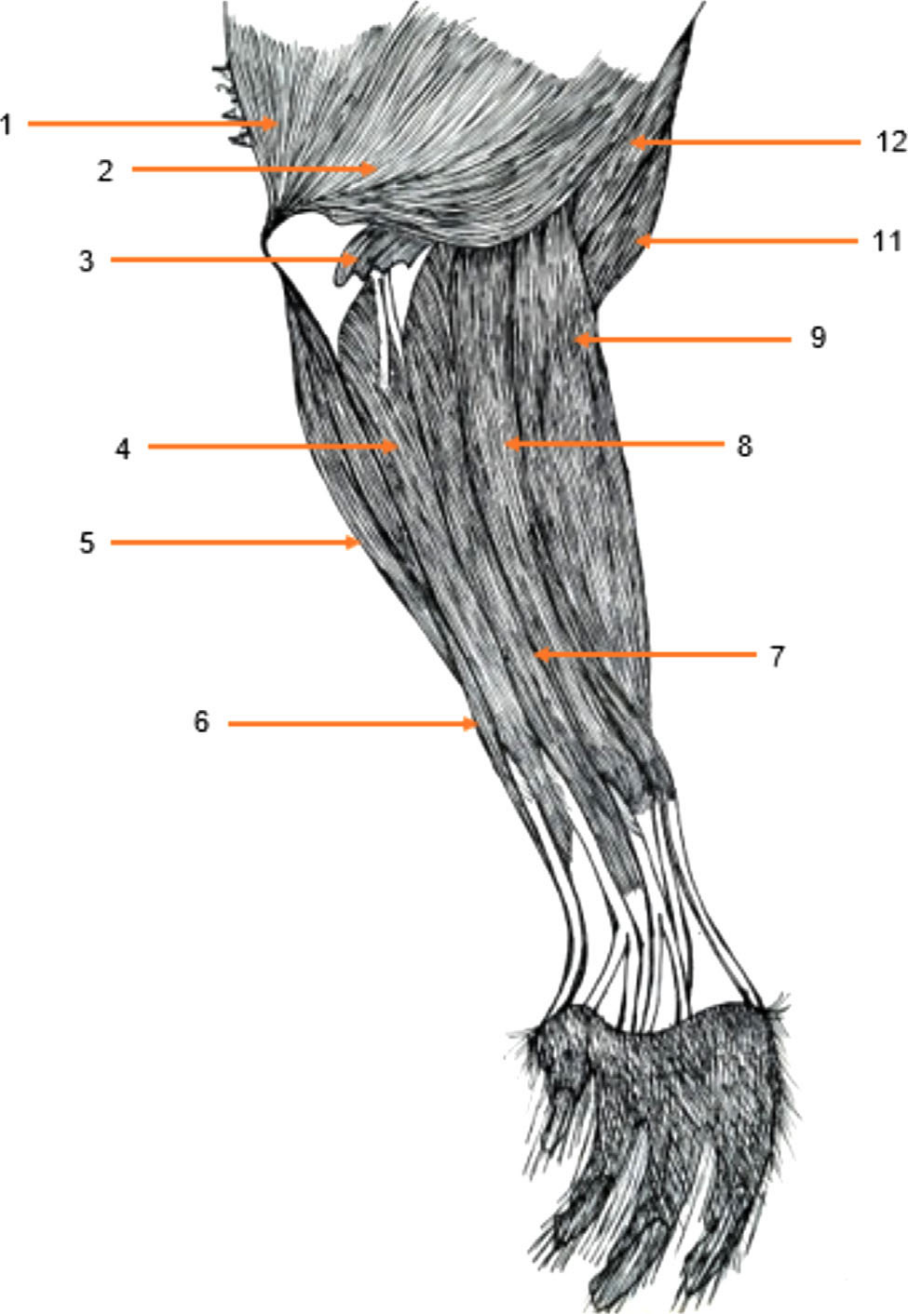
Muscles of the forearm of the aulacod (right limb – lateral aspect): 1: long head of the triceps brachii muscle, 2: lateral head of the triceps brachii muscle, 3: anconus muscle, 4: lateral ulnar muscle, 5: ulnar head of the ulnar flexor carpi muscle, 6: humeral head of the ulnar flexor carpi muscle, 7: extensor oblique muscle of the carpus, 8: lateral extensor muscle of the fingers, 9: common extensor muscle of the fingers, 10: radial extensor muscle of the carpus, 11: biceps brachii muscle, 12: brachial muscle

##### Tensor muscle of the antebrachial fascia

This muscle has not been observed in the aulacode.

### 3.3 Muscles of the forearm

#### 3.3.1 Dorsal antebrachial muscles (Figures 3, 4, 5)

##### Extensor carpi radialis muscle

It is quite thick and voluminous. This double muscle is united into one. It attaches to the radius and its lower tendon reaches the metacarpal of the thumb.

**FIGURE 4:**
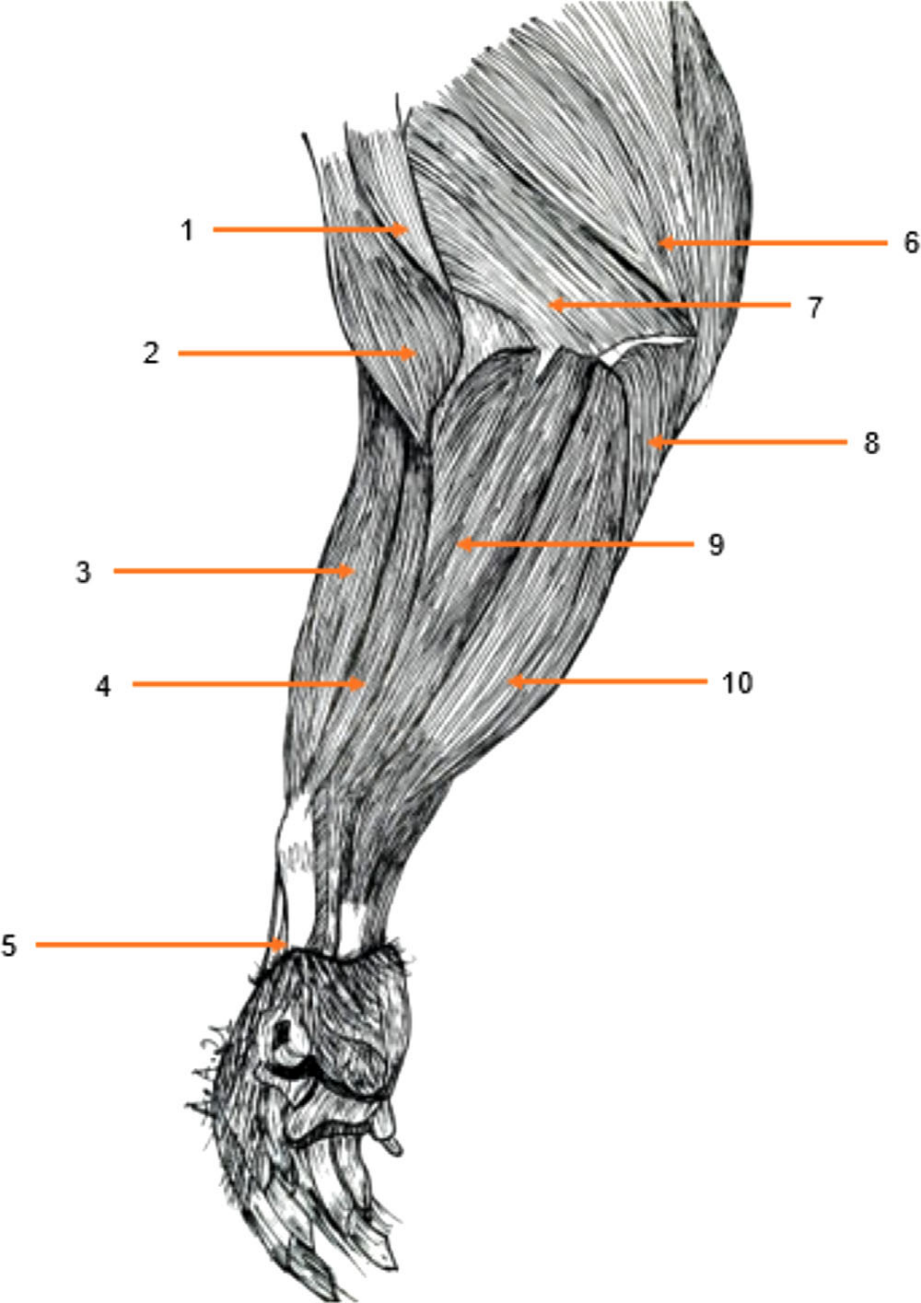
Muscles of the forearm of the aulacod (right limb – medial side): 1: coracobrachial muscle, 2: biceps brachii muscle, 3: radial extensor carpi muscle, 4: round pronator muscle, 5: oblique extensor carpi muscle, 6: long head of triceps brachii muscle, 7: medial head of triceps brachii muscle, 8: ulnar flexor carpi muscle, 9: radial flexor carpi muscle, 10: superficial flexor carpi muscle

**FIGURE 5:**
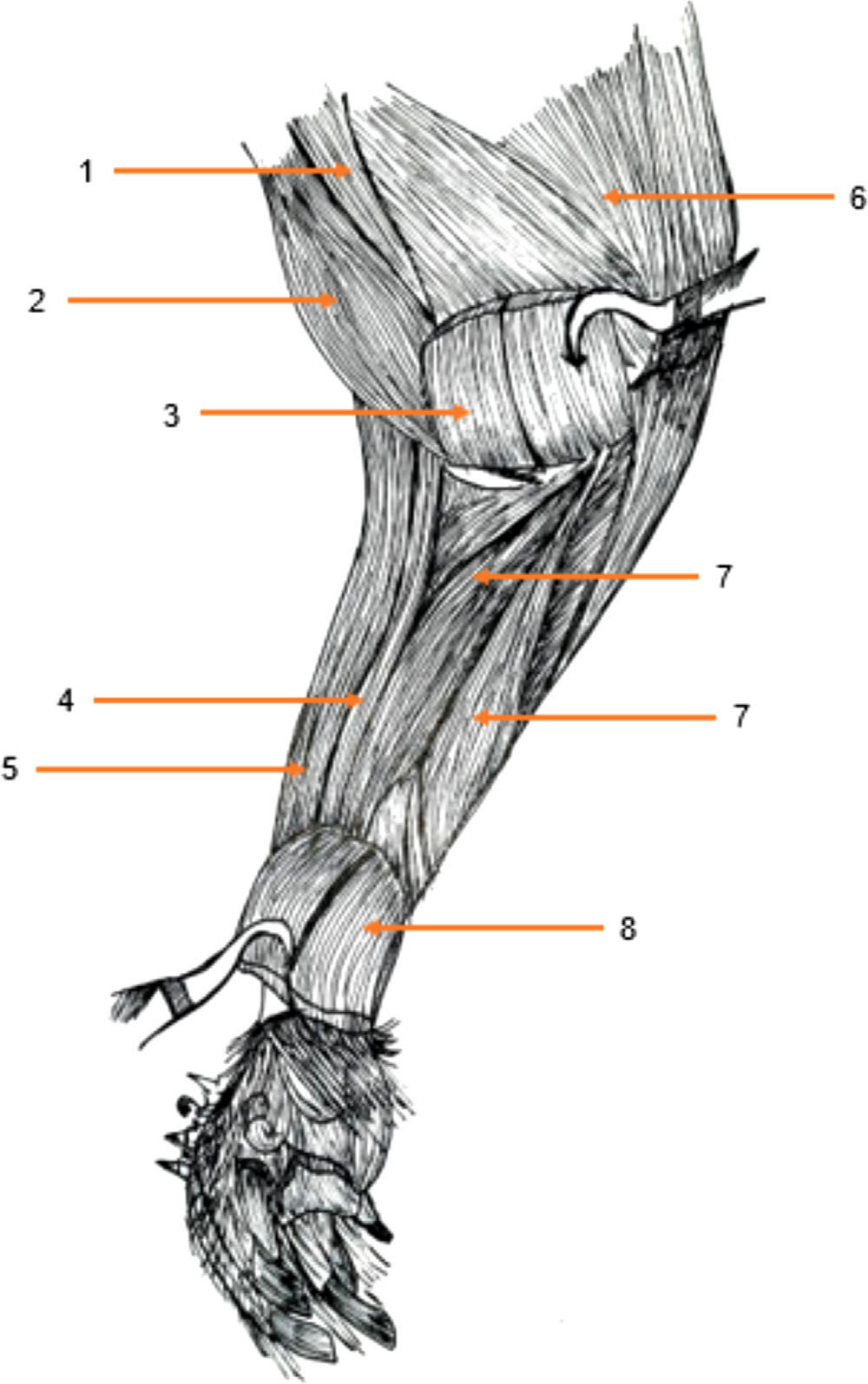
Muscles of the forearm of the aulacod (right limb – medial aspect – deep plane) after cutting radial flexor carpi muscle and superficial flexor carpi muscle: 1: coracobrachial muscle, 2: biceps brachii muscle, 3: muscle portion of the flexor carpi radialis muscle, 4: extensor carpi radialis muscle, 5: pronator pollicis muscle, 6: triceps brachii muscle, 7: flexor carpi profundus muscle, 8: muscle portion of flexor carpi superficialis muscle

##### Extensor digitorum communis muscle

This muscle has a single fusiform fleshy body, continued by several tendons joined at their origin. The muscle is partially covered by the extensor carpi radialis muscle, to which it adheres at its origin. The tendon becomes distinct towards the distal third of the forearm and it is apparently simple, but can be cleaved into four branches, each of which receives a part of the fleshy body. The muscle originates on the distal end of the epicondylar ridge of the humerus and on the base of the lateral epicondyle and inserts on the extensor process of the distal phalanx.

##### Lateral extensor muscle of the digits

It shows a slender appearance and divides distally into two portions each extended by a tendon to the proximal end of the first phalanx of the two outer digits. The fleshy body arises on the lateral collateral ligament of the elbow and on the adjacent tuberosity of the radius. It attaches to the lateral epicondyle of the humerus and inserts by its distal tendon to the proximal end of the fifth metacarpal, after having attached to the supracarpal bone. This muscle extends the finger or fingers on which it inserts.

##### Extensor carpi obliquus muscle

It is a single muscle with a fleshy portion and a distal tendon. The body is fleshy, flat, thin and moulded to the dorsal aspect of the radius. It originates along the radioulnar joint. Its inferior tendon crosses obliquely the tendons of the extensor carpi radialis muscle and inserts on the proximal end of the thumb metacarpal bone.

##### Lateral ulnar muscle

This muscle is spindle‐shaped, flattened from one side to the other (lateral to medial). The proximal tendon originates on the top of the lateral epicondyle of the humerus and the muscle inserts on the proximal edge of the pisiform bone. This muscle is flexor of the carpus and the hand

#### 3.3.2 Palmar antebrachial muscles (Figures 3, 4, 5)

##### Ulnar flexor carpi muscle

This muscle has two elongated parts, joined distally to end in a single tendon. The caput humerale is fusiform and flattened. But the caput ulnare is weak and thin. The caput humerale originates at the base of the medial epicondyle, caudal to the flexor carpi radialis muscle, and the caput ulnare at the caudal edge of the olecranon. It is a flexor of the carpus and of the hand.

##### Flexor carpi radialis muscle

It is formed by a fleshy body between two tendons. The fleshy body is fusiform, broad and flat. The proximal tendon is very short. The distal tendon is much longer, thin and cylindroid. Its proximal tendon originates at the base of the medial epicondyle of the humerus, between the medial collateral ligament of the elbow and the ulnar flexor carpi muscle. The muscle inserts at the base of the medial epicondyle of the humerus and inserts on the proximal end of the metacarpal bone III. The muscle flexes the carpus and the whole hand on the forearm.

##### Superficial flexor muscle of the digits

Its fleshy body is elongated, prismatic and flattened in a dorsopalmar direction. The tendon is initially single, then divides at the level of the carpus into four branches that diverge only in the metacarpus of the four external digits, respectively. It originates at the top of the medial epicondyle by a very short tendon.

##### Muscle flexor digitorum (digitalis) profundus

It has three parts. The caput humerale is large and fusiform. It comes from a very short and very strong tendon whose origin is linked to that of the superficial flexor muscle of the digitis. The caput ulnare is weaker in the aulacode and the caput radiale is a long flexor muscle of the thumb, more voluminous than the caput ulnare. The caput ulnare is attached to the greater length of the ulna.

##### Pronator round muscle

It is a thin, narrow band superimposed on the medial collateral ligament of the elbow. It originates at the base of the medial epicondyle of the humerus and inserts on the medial border of the radius. It is pronator and can in addition contribute to the flexion of the forearm on the arm.

## 4 DISCUSSION

The muscles of the thoracic limb are grouped around the girdle and the various rays of this limb. They constitute four major groups, each of which corresponds to a natural region: shoulder, arm, forearm and hand. The muscles of each of these groups act on the following segment: those of the shoulder mobilise the arm; those of the arm move the forearm; the antebrachial muscles move the hand; as for the muscles of the hand, they move the digits in relation to each other and are only well developed in polydactyl species. Since the latter show almost no differences from other rodents of the same family, we focused on the scapular, brachial and antebrachial muscles.

In the lateral scapular region, the deltoid muscle in the aulacode presents similarities with that of rodents in general and in particular with that of the guinea pig. Indeed, the three parts are unequal and distinct in the guinea pig (Alezais, 1900), and the aulacode. However, they are joined in the aulacode. In the latter, the acromial part and the clavicular part of the deltoid muscle have a more or less triangular shape. In contrast, in the guinea pig, the clavicular part is thin and flattened (Alezais, 1900). The spinal part of the deltoid muscle is small and leaves along the scapular spine as in the guinea pig and rabbit. In the hare, however, the three portions are distinct and have the acromial portion of the deltoid muscle smaller than the others (Alezais, 1900). The small round muscle has not been observed in the aulacode because it is either hidden by the size of the deltoid muscle or because it appears to be absent in rodents as Lesbre (1907) stated in the porcupine and (Alezais, 1900) in the guinea pig (Alezais, 1900, Lesbre, 1907). However, the petty round muscle is short and narrow in the rabbit (Barone, 2000), and in the hare (Alezais, 1900) where it seems to be reduced to the role of tensor of the infraspinous fascia. The supraspinous and infraspinous muscles in the aulacode do not have any specific characteristics. They resemble those of the rabbit and the guinea pig.

In the medial scapular region, the subscapularis muscle is voluminous in the aulacode but rather thick and relatively narrow in the rabbit (Barone, 2000). As for the greater muscle, it is large and thick in both the aulacode and the rabbit. In fact, the round muscle is less voluminous than the dorsalis major muscle in the aulacode, and in the rabbit (Barone, 2000).

The coracobrachial muscle shows a broad and strong appearance as in the rabbit (Barone, 2000). It is arranged in both species in the same way and has, in addition to its middle part in the aulacode, a superior or proximal part, which is not the case in the guinea pig (Alezais, 1900). In the cranial brachial region, the biceps brachii muscle is fusiform as in the porcupine epicondyle (Lesbre, 1907) while that of the rabbit is cylindroid (Barone, 2000). The brachial muscle is fusiform and long throughout the caudal part of the aulacode humerus. This appearance is different in the rabbit, which has a broad, strong muscle terminating on the ulna (Barone, 2000).

In the caudal brachial region, the triceps brachii muscle has little morphology variation in rodents in general (Alezais, 1900). In the aulacode, all three parts exist and generally present a long and strong appearance as in the rabbit (Barone, 2000). Indeed the caput laterale is wide and the caput mediale is also long as in the rabbit (Barone, 2000). The caput longum is large in the aulacode unlike that of the rabbit which presents a thick but narrow and fusiform appearance, much like in the cat (Barone, 2000).

The anconeal muscle is short, flattened and located laterally in the aulacode, but this anconeal muscle is not very distinct as in the rabbit, which has a well isolated medial anconeal muscle as well. The tensor muscle of the antebrachial fascia is a narrow band in the rabbit (Barone, 2000), but we have not observed it in the aulacode. Indeed, Alezais (1900) and Lesbre (1907) did not describe this muscle in the guinea pig and the porcupine respectively, both of which are rodents.

The dorsal antebrachial muscles show the extensor carpi radialis, extensor digitorum communis, extensor digitorum lateralis, extensor digitorum obliquus and extensor digitorum. Unlike some rodents, the long supinator or brachioradialis muscle has not been observed in the aulacode in the dorsal region of the forearm. Indeed, this muscle has been reported absent in the porcupine (Lesbre, 1907), the guinea pig (Alezais, 1900) and the rabbit (Barone, 2000). The radial extensor muscle of the digits is quite thick, voluminous and normally doubled and fused in the aulacode and in the guinea pig (Alezais, 1900), whereas in the rabbit (Barone, 2000) and in the hare (Alezais, 1900), this muscle is split into two tendons in its distal part. In the aulacode, the common extensor muscle of the digits presents similarly in the hare (Alezais, 1900) and in the rabbit (Barone, 2000), a single fusiform fleshy body, continued by several tendons joined at their origin. The lateral extensor muscle of the digits is small and is identical to that of the porcupine epicondyle (Lesbre, 1907) and the rabbit (Alezais, 1900). Apart from its thinness, the extensor carpi obliquus muscle consists of a single part in the aulacode like the extensor carpi obliquus muscle of the rabbit (Barone, 2000). The lateral ulnar muscle is fusiform, but is arranged as in carnivores and rabbits.

In the palmar region, the ulnar flexor carpi muscle shows the caput humerale fusiform and flattened, while the caput ulnare is weak and thin. According to Barone (2000), the rabbit has a relatively thick caput ulnare that partially covers the caput humerale. The flexor carpi radialis muscle is formed by a broad, flat, fusiform fleshy body, as in the rabbit, but it is small in the latter (Barone, 2000). The superficial flexor muscle of the digits is elongated, prismatic, flattened in the dorsopalmar direction and has the same arrangement as in the guinea pig (Alezais, 1900) and the rabbit (Barone, 2000). However, it is weak in the porcupine epicondyle (Lesbre, 1907). The deep flexor muscle of the digits shows the caput humerale mainly bulky and fusiform. The caput radiale is also quite large in the aulacode but the caput ulnare is the weakest. These aspects seem to be identical in rabbits and carnivores (Barone, 2000). We have not observed a pronator quadratus muscle in the aulacode, but according to Alezais (1900), this muscle exists in the aulacode where it occupies the entire length of the contiguous edges of the radius and ulna. Like Barone (2000), he also points out that in the guinea pig, the hare and the rabbit, this muscle is missing.

## 5 CONCLUSION

Our study on the descriptive anatomy of the muscles of the thoracic limb of the aulacode, the comparison of the thoracic limb of the aulacode with other rodents such as the guinea pig (*Cavia porcellus*), the epic pig (*Hystrix indica*) and lagomorphs such as the rabbit (*Oryctolagus cuniculus*) and the hare showed analogies and differences. Thus, the deltoid muscle is very developed with its three portions. The other muscles of the scapula show many similarities with those of the rabbit; however, they are stronger in the aulacode. The arm shows fusiform biceps brachii and brachii muscles like those of the porcupine (*Histrix cristata*). The triceps brachii muscle has a very bulky long part (caput longum). The appearance of the forearm muscles (extensors and flexors) is generally more prominent than in other species.

## AUTHOR CONTRIBUTIONS

This work was carried out in collaboration among all authors. All authors read and approved the final manuscript.

## CONFLICT OF INTEREST

The authors declare no conflict of interest.

## ETHICAL APPROVAL

It is not applicable.

### PEER REVIEW

The peer review history for this article is available at https://publons.com/publon/10.1002/vms3.729

## Data Availability

The data that support the findings of this study are available from the corresponding author upon request
